# Noninvasive diagnosis model for predicting significant liver inflammation in patients with chronic hepatitis B in the immune-tolerant phase

**DOI:** 10.1038/s41598-025-87756-4

**Published:** 2025-01-24

**Authors:** Shanshan Chen, Lu Huang, Yili Chu, Jiangshan Lian, Hui Shao, Tingting Wang, Xuehan Zou, Haijun Huang

**Affiliations:** 1 Emergency and Critical Care Center, Department of Emergency Medicine, Zhejiang Provincial People’s Hospital (Affiliated People’s Hospital, Hangzhou Medical College), Hangzhou, 310014 Zhejiang China; 2 Center for General Practice Medicine, Department of Infectious Disease, Zhejiang Provincial People’s Hospital (Affiliated People’s Hospital, Hangzhou Medical College), Hangzhou, 310014 Zhejiang China; 3 State Key Laboratory of Infectious Diseases, Department of Infectious Disease, The First Affiliated Hospital, National Medical Center for Infectious Diseases, Zhejiang University School of Medicine, Zhejiang University, Hangzhou, 310003 China; 4 Department of Infection, Zhejiang Taizhou Hospital Affiliated to Wenzhou Medical University, Taizhou, 317000 China; 5 No. 158 Shangtang Road, Hangzhou City, Zhejiang Province China

**Keywords:** Chronic hepatitis B, Immune-tolerant phase, Nomogram, Noninvasive diagnosis model, Liver inflammation, Gastrointestinal diseases, Diseases, Gastroenterology

## Abstract

The presence of significant liver inflammation is an important indication for antiviral therapy in immune-tolerant (IT)phase with chronic hepatitis B(CHB) patients. This study aims to establish a non-invasive model to assess significant liver inflammation in the IT-phase of CHB patients. This multicenter retrospective study included a total of 535 IT-phase CHB patients who underwent liver biopsy, and were randomly divided into a training and a validation set. In the training cohort, the relevant indices were initially screened using univariate analysis. Then the least absolute shrinkage and selection operator and multivariable logistic regression were used to identify the significant independent risk factors and establish a predictive model. A diagnostic nomogram was constructed. Calibration curves, decision curve analysis, and receiver operating characteristic curves were utilized to evaluate the performance of the nomogram. In this study, 37.0% of the patients exhibited significant liver inflammation. Baseline characteristics revealed a median age of 35.0 years, with males accounting for 51.7% of the cohort. Age, Aspartate aminotransferase (AST), Prothrombin (PT), Albumin (ALB) and Hepatitis B virus DNA (HBV DNA) were identified as independent predictors of significant liver inflammation in the immune-tolerant phase, and a nomogram was constructed based on these indicators. The predictive model demonstrated good calibration and discrimination in both the training set and the validation set (aera under the curve (AUC) of 0.741 and 0.740, respectively). The nomogram can accurately identify significant liver inflammation in immune-tolerant phase CHB patients and facilitate the early initiation of antiviral therapy, thereby reducing the need for clinical liver biopsies.

**Supplementary Information:**

The online version contains supplementary material available at 10.1038/s41598-025-87756-4.

## Introduction

Hepatitis B virus (HBV) infection remains a serious global public health issue, affecting approximately 257 million people. Each year around 887,000 people die from HBV-related diseases, particularly cirrhosis and hepatocellular carcinoma (HCC)^1–3^. It is estimated that more than 50 million individuals are in the immune-tolerant (IT) phase. Typically, the treatment of chronic hepatitis B (CHB) focuses primarily on patients with immune-active (IA) disease, cirrhosis, and decompensated cirrhosis. However, the management of IT-phase CHB mainly relies on regular monitoring rather than antiviral therapy^4^.

There is controversy regarding the severity of liver damage and the necessity of antiviral therapy in the IT phase. The EASL, AASLD and APASL guidelines suggest that the definition and management of IT-phase patients are not entirely consistent^4–6^. The IT-phase is primarily defined based on serum hepatitis B virus DNA (HBV DNA), serum Alanine aminotransferase (ALT) levels and liver histological characteristics. While HBV DNA is an important marker for assessing disease progression and treatment indications, there is still controversy regarding the relationship between HBV DNA levels and disease severity^7–10^. Additionally, ALT levels have long been considered a common indicator for evaluating liver inflammation and determining the timing for initiating antiviral therapy^6–11^. However, studies, including our previous research, have shown that significant liver inflammation can be present in CHB patients with normal ALT levels, indicating that ALT levels do not always correlate with the severity of liver damage^12,13^.

Although liver biopsy is considered the “gold standard” for assessing liver histology, its invasive nature (including sampling error, high cost and associated risks) makes it difficult to perform routinely in clinical practice^6^. As a result, numerous non-invasive models have been developed to evaluate the severity of liver histology, including the aspartate aminotransferase to platelet ratio index (APRI) and the fibrosis-4 (FIB-4) index^14,15^. Typically, these methods have high accuracy for assessing liver fibrosis^16,17^. However, there are relatively few non-invasive models for evaluating significant liver inflammation in CHB patients, particularly those in the IT-phase. Therefore, there is an urgent need to develop a non-invasive model to assess liver inflammation in IT-phase patients.

This study aimed to establish and validate a non-invasive nomogram model to predict significant liver inflammation in IT-phase CHB patients, facilitating the early initiation of antiviral therapy.

## Methods

We retrospectively included 535 CHB patients who received liver biopsy in Zhejiang Provincial People’s Hospital, Zhejiang Taizhou Hospital Affiliated to Wenzhou Medical University and The First Affiliated Hospital, Zhejiang University School of Medicine, Zhejiang University from January 2014 to December 2022. We confirm that all methods were performed in accordance with relevant guidelines and regulations. Chronic HBV infection was defined as being seropositive for hepatitis B surface antigen for at least 6 months^18^. Inclusion criteria: the inclusion criteria for all patients were based on the EASL and AASLD guidelines, including positive hepatitis B surface antigen for more than six months; positive hepatitis B e antigen; HBV DNA level higher than 10^6^ IU/ml, and normal ALT (40U/L), all of patients had not undergone antiviral therapy. Exclusion criteria included: hepatitis C virus (HCV) infection, hepatitis D virus (HDV) infection, hepatitis E virus or human immunodeficiency virus (HIV) co-infection and other causes of liver disease, alcoholic liver disease, non-alcoholic fatty liver disease, autoimmune liver disease, decompensated cirrhosis, HCC, insufficient liver biopsy samples and incomplete clinical data. This study was approved by the Ethics Committee of Zhejiang Provincial People’s Hospital.

All patients underwent ultrasound-guided percutaneous liver biopsy. Liver biopsy was performed with an 18G biopsy needle. The biopsy specimens were fixed with formalin, embedded with conventional paraffin, and stained with hematoxylin-eosin (HE). The specimen was at least 1.5 cm in length and contained at least 6 complete portal vein bundles. Necrotizing inflammation (G0-4) was histologically graded according to the Scheuer classification system^19^. All slides were evaluated independently by two pathologists and without knowledge of the patient’s clinical data. According to liver histological changes, patients were divides into mild group (G<2) and moderate to severe group (G ≥ 2).

Demographic data and laboratory indicators were collected before liver biopsy. Including age, sex, white blood cell (WBC), platelet count (PLT), prothrombin time (PT), International Standardized Ratio (INR), albumin (ALB), globulin (GLB), ALT, aspartate aminotransferase (AST), glutamyl transpeptase (GGT), Alkaline phosphatase (ALP) and serum total bilirubin (TBIL). Hepatitis B surface antigen(HBsAg), hepatitis B surface E antigen(HBeAg) and core antibody were detected by CLIA system. Real-time polymerase chain reaction system (ABI7300; Foster City, CA Applied Biosystems) to detect serum HBV-DNA levels. Normal experimental values are as follows: ALT ≤ 40U/L. We confirm that informed consent was obtained from all subjects and their legal guardian.

The definition of the IT phase according to the AASLD is as follows: (a) positive hepatitis B surface antigen for more than six months; (b) positive hepatitis B e antigen; (c) HBV DNA level higher than one million IU/mL and (d) normal (35 U/L for males and 25 U/L for females) or minimally elevated ALT^11^. The definition of the IT phase according to the EASL is as follows: (a) positive hepatitis B surface antigen for more than six months, (b) positive hepatitis B e antigen; (c) HBV DNA level higher than 107IU/mL, and (d) normal ALT (40U/L)^4^.

### Statistical analysis

Data analyses were analyzed using SPSS (version 26.0, IBM, NY) and R (version 4.2.0, Vienna, Austria). First, we randomly divided into training and validation sets at a ratio of 3:2, and the variables were compared. Non-normal data were presented as median (interquartile ranges). In the univariate analysis, continuous variables were compared using the Student’s t-test (normal distribution) and Mann-Whitney U test (skewed distribution), which were presented as mean ± standard deviation and median (interquartile range, IQR), respectively. Categorical variables were presented as number (percentage) and compared by the chi-square test or Fisher’s exact test. In the training sets, the least absolute shrinkage and selection operator (LASSO) regression analysis were used for multivariate analysis to screen independent risk factors for significant liver inflammation. Then, variables obtained from the LASSO regression were included in the logistic regression. Based on these results, a nomogram was constructed. The performance of the nomogram was verified by the receiver operating characteristic (ROC) curve, decision curve analysis (DCA) and calibration curve. *P* < 0.05 with two sides considered statistical significance.

## Results

The study included CHB patients diagnosed via liver biopsy and from the Infectious Disease Departments of Zhejiang Provincial People’s Hospital, Taizhou Hospital and the First Affiliated Hospital of Zhejiang University between January 2014 and December 2022. Based on inclusion and exclusion criteria, 535 IT phase CHB patients were finally included. Computer-generated random method was used to select 60% of the patients as the training cohort and 40% as the validation cohort.

The baseline characteristics of the patients are shown in Table 1. The median age was 35.0 and 36.0 years, with 159 males (49.4%) and 114 males (53.5%) in the training and validation cohort. Significant liver inflammation (G ≥ 2) was observed in 119(37.0%) and 79(37.1%) patients with IT-phase in two cohort.

**Table 1:** Characteristics of patients with chronic hepatitis B in the immune tolerance phase.

Characteristics	Training set *N* = 322	Validation set *N* = 213	*P* value
Age(years)	35.0(29.0–43.0)	36.0(29.0–44.0)	0.218
Sex (%)			0.552
Male	159(49.4)	114(53.5)	
PT (s)	11.9(11.3–12.8)	11.9(11.3–12.7)	0.968
INR	1.01(0.97–1.07)	1.02(0.97–1.07)	0.547
WBC (×10^9^/L)	5.7(4.8–6.9)	5.5(4.6–6.6)	0.221
Hb (×10^9^/L)	142.0(131.2–153.0)	143.0(131.0-156.0)	0.961
PLT (×10^9^/L)	201.0(167.2-234.8)	192.0(160.0-231.0)	0.321
ALB (g/L)	43.8(40.8–46.1)	44.1(41.4–46.5)	0.391
GLB (g/L)	27.9(24.9–30.6)	28.0(25.4–30.3)	0.501
ALT (U/L)	26.0(20.0-33.8)	27.0(20.0–33.0)	0.598
AST (U/L)	24.0(20.0–30.0)	24.0(21.0–29.0)	0.253
GGT (U/L)	17.0(14.0–24.0)	17.0(14.0–24.0)	0.406
ALP (U/L)	71.0(59.0–87.0)	71.0(60.0–89.0)	0.988
TBIL (umol/L)	13.0(10.0-16.6)	12.1(9.6–16.0)	0.969
HBVDNA (Log_10_ IU/ml)	7.9(7.3–8.5)	7.8(7.0-8.3)	0.105
Liver inflammation grade (%)			0.978
G0	5(1.5)	5(2.3)	
G1	198(61.5)	129(60.6)	
G2	101(31.4)	67(31.5)	
G3	15(4.7)	10(4.7)	
G4	3(0.9)	2(0.9)	
Fibrosis stage (%)			0.978
S0	93(28.9)	64(30.1)	
S1	142(44.1)	94(44.1)	
S2	67(20.8)	41(19.2)	
S3	12(3.7)	8(3.8)	
S4	8(2.5)	6(2.8)	

PT: Prothrombin; INR: International normalized ratio; WBC: White blood cell; PLT: Platelet; ALB: Albumin; GLB: Globulin; ALT: Alanine aminotransferase; AST: Aspartate aminotransferase; GGT: Glutamyl transpeptidase; ALP: Alkaline phosphatase; TBIL: Total bilirubin; HBV DNA, Hepatitis B virus DNA;

We further compared the clinical characteristics and laboratory data of immune-tolerant patients with and without significant liver inflammation in the training cohort. There were significant differences between mildly (G<2) and significantly (G ≥ 2) liver inflammation in terms of Age, PT, HB, PLT, ALB, ALT, AST, GGT, ALP and HBV DNA (all *P*<0.05) (Table 2).

**Table 2:** The laboratory and characteristics of patients with and without significant liver inflammation in the training cohort.

Characteristics	G<2 *N* = 203	G ≥ 2 *N* = 119	*P* value
Age(years)	34.0(28.0-40.5)	39.0(30.0–45.0)	<0.001
Sex (%)			0.099
Male	109(53.7)	50(42.0)	
PT (s)	11.8(11.1–12.5)	12.3(11.7–13.1)	<0.001
INR	1.01(0.96–1.05)	1.02(0.98–1.09)	0.698
WBC (×10^9^/L)	5.8(4.9–6.9)	5.6(4.7–6.7)	0.520
Hb (×10^9^/L)	143.0(133.0-157.0)	140.0(129.5–149.0)	0.006
PLT (×10^9^/L)	202.0(174.0-232.5)	188.0(153.5–235.0)	0.025
ALB (g/L)	44.4(41.7–46.8)	42.5(39.8–45.1)	<0.001
GLB (g/L)	27.3(24.7–29.9)	29.2(25.9–32.5)	0.542
ALT (U/L)	25.0(19.0–31.0)	27.0(21.5–35.0)	0.007
AST (U/L)	23.0(19.5–27.0)	28.0(23.0–35.0)	<0.001
GGT (U/L)	16.0(12.0–21.0)	18.0(15.0-29.5)	0.002
ALP (U/L)	69.0(58.0–82.0)	77.0(62.0–97.0)	0.001
TBIL (umol/L)	13.0(10.0-16.7)	13.0(9.9–16.6)	0.895
HBVDNA (Log_10_ IU/ml)	8.1(7.6–8.5)	7.4(6.6–8.2)	<0.001
APRI	0.28(0.23–0.36)	0.35(0.28–0.57)	<0.001
FIB-4	0.81(0.58–1.07)	1.01(0.70–1.76)	<0.001

**Fig. 1:**
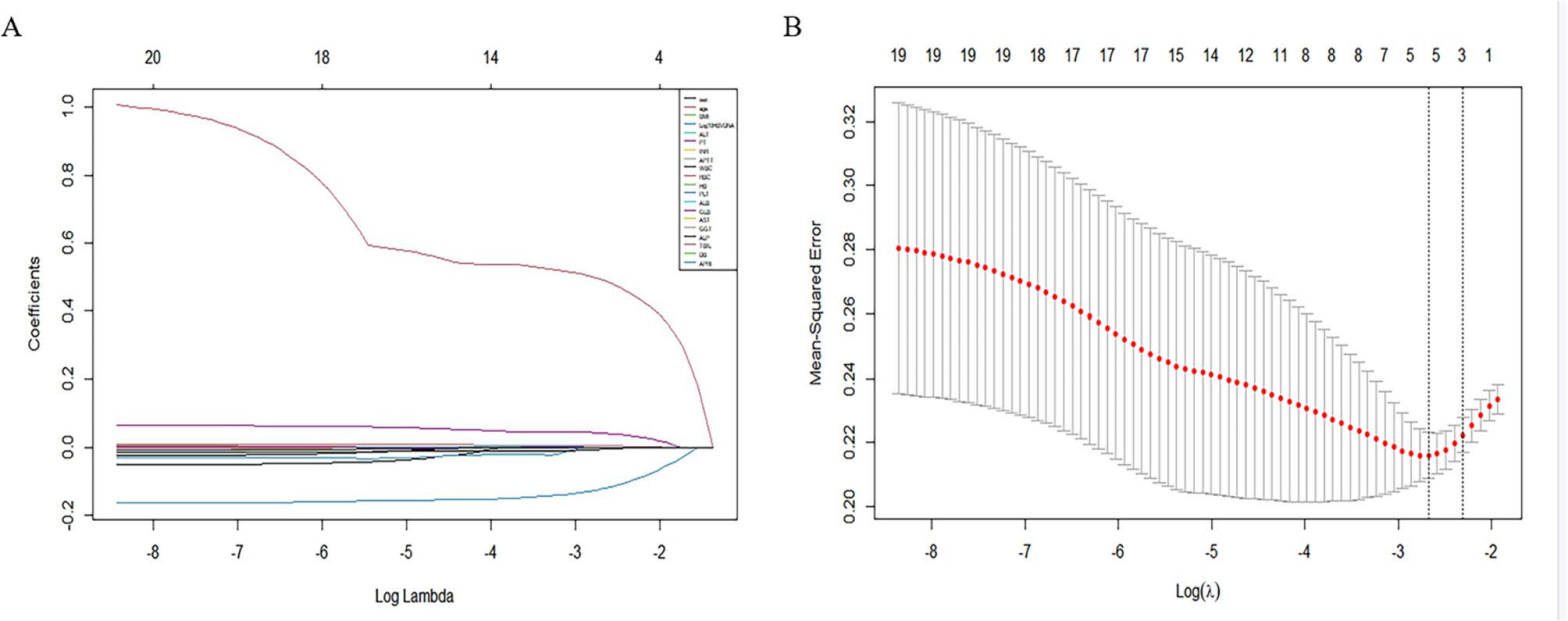
Predictor selection using LASSO regression analysis. (A) three predictors were selected by deriving the optimal λ. (B) the optional penalty coefficient λ of LASSO regression was determined by 10-fold cross-validation and minimum criterion.

**Fig. 2:**
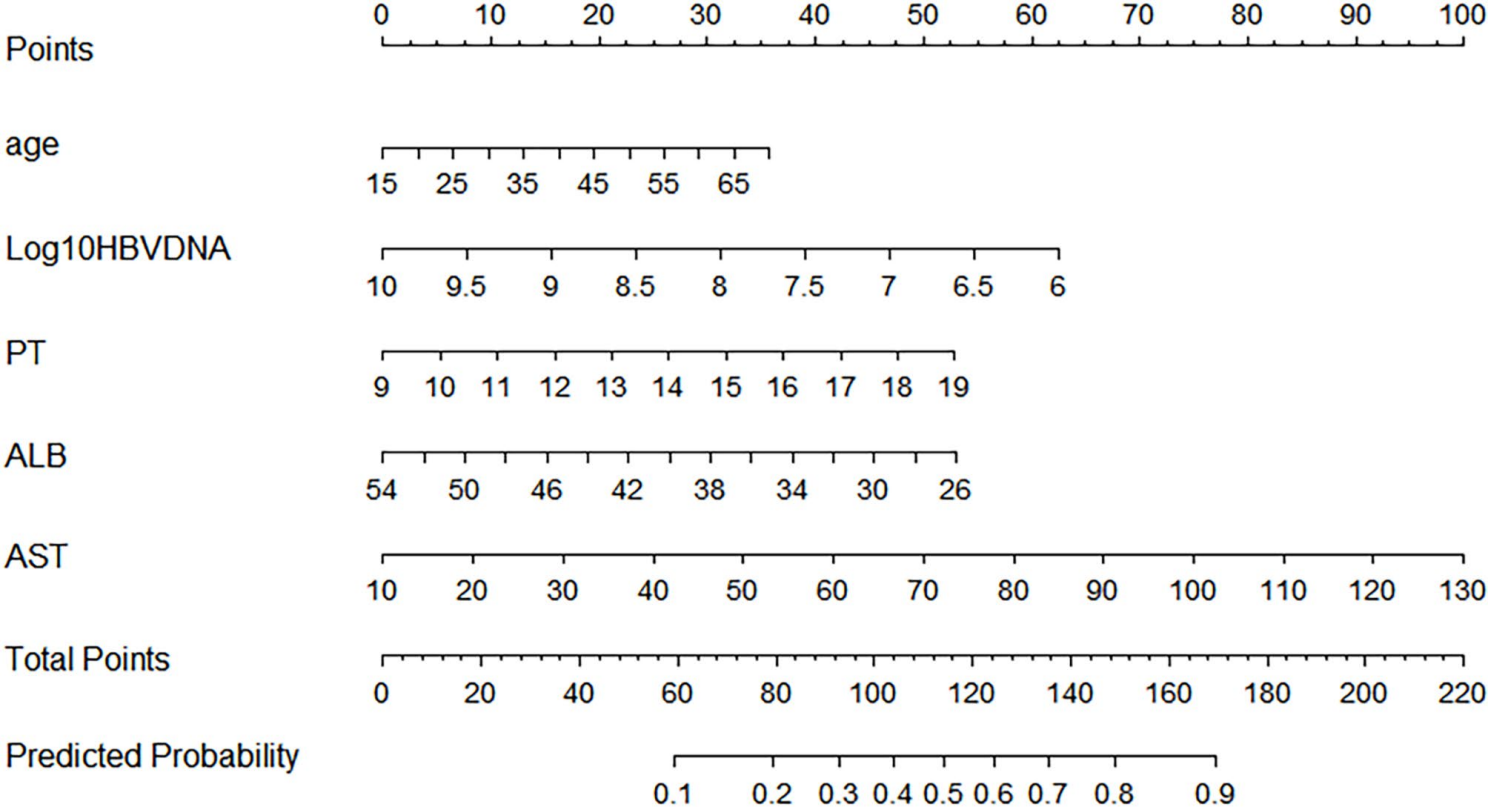
A nomogram for the prediction of significant liver inflammation in patients with chronic hepatitis B in the immune tolerate phase.

PT: Prothrombin; INR: International normalized ratio; WBC: White blood cell; PLT: Platelet; ALB: Albumin; GLB: Globulin; ALT: Alanine aminotransferase; AST: Aspartate aminotransferase; GGT: Glutamyl transpeptidase; ALP: Alkaline phosphatase; TBIL: Total bilirubin; HBV DNA, Hepatitis B virus DNA; APRI, Aspartate aminotransferase to platelet ratio index; FIB-4, Fibrosis index based on the 4 factors;

In the training cohort, LASSO regression was used to select parameters for assessing significant liver inflammation, with the coefficient curves shown in Fig. 1A. The cross-validation error plot for the LASSO regression model is displayed in Fig. 1B. Five predictive variables (Age, AST, PT, ALB and HBV DNA) were ultimately selected from 17 candidate variables as being positively correlated with significant liver inflammation. These factors were incorporated into a multivariable logistic regression to establish a nomogram for predicting significant liver inflammation during the IT phase, referred to as the nomogram (Fig. 2). The total score is calculated by summing the scores of all predictive factors to determine the risk probability of significant liver inflammation in CHB patients in the immune-tolerant phase.

**Fig. 3:**
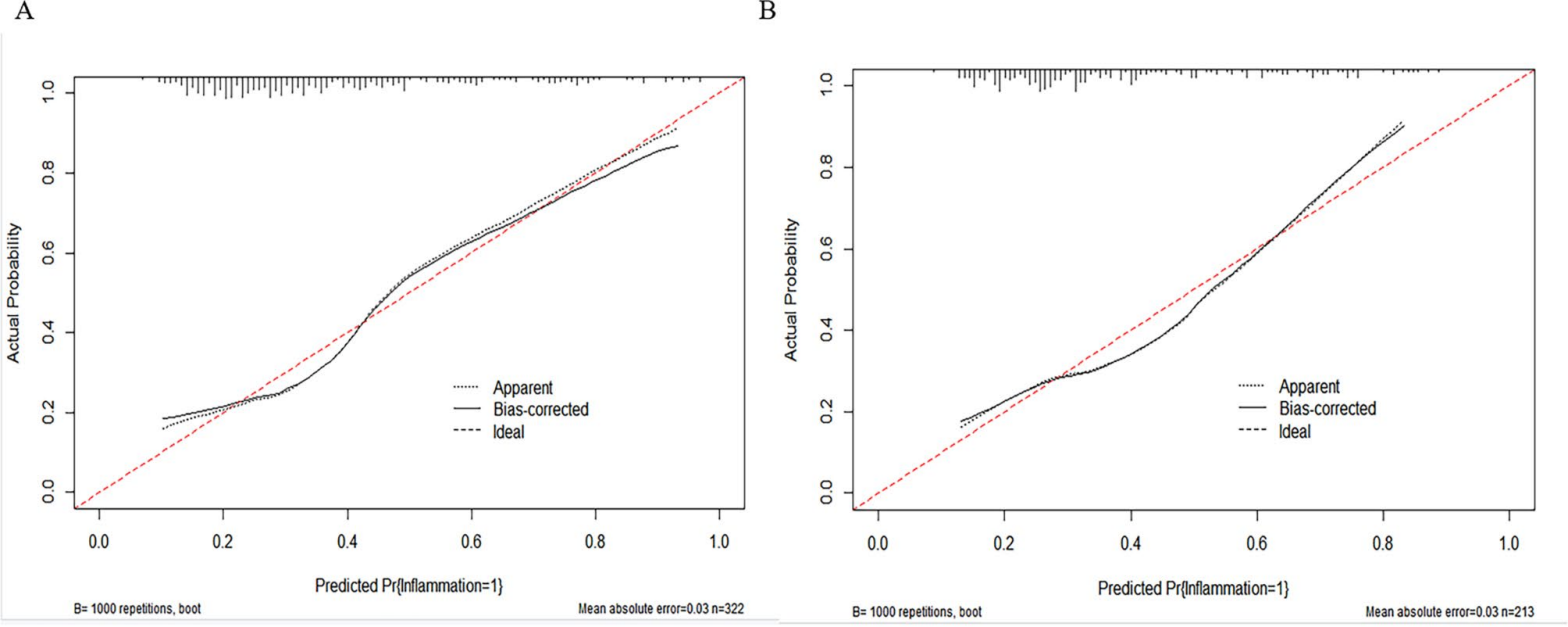
Calibration curves of the model for predicting significant liver inflammation in the training set (A) and validation set (B). The x-axis and y-axis represent the predicted and actual probability of significant liver inflammation, respectively.

**Fig. 4:**
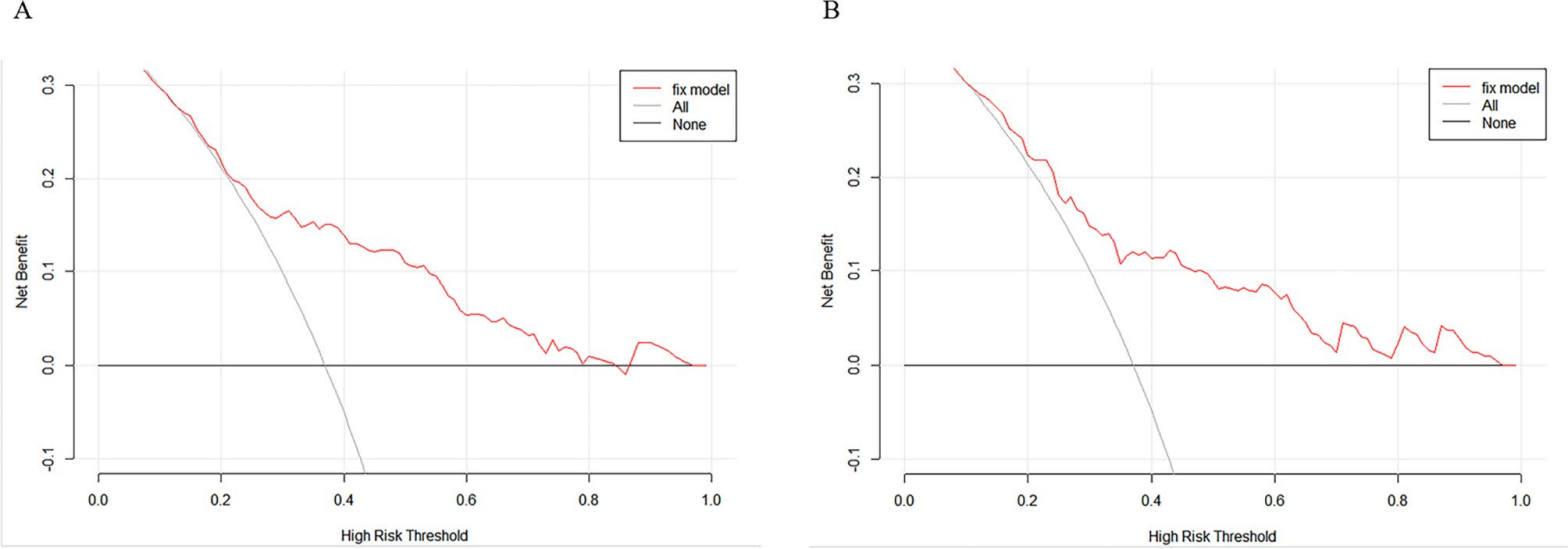
Decision curve analysis for the prediction of significant liver inflammation in the training cohort (A) and validation cohort (B). The x-axis is the threshold probability, the y-axis is the net benefit.

The nomogram was calibrated using the Hosmer-Lemeshow test and calibration plots with 500 bootstrap resamples, indicating good consistency between the predicted probabilities and the observed probabilities of significant liver inflammation (Fig. 3). Additionally, DCA showed that the nomogram had a threshold probability range for distinguishing significant liver inflammation in CHB patients in the IT-phase (Fig. 4). The clinical impact curve results demonstrated that the model’s predicted values were moderately well-aligned with the true positive rates.

**Fig. 5:**
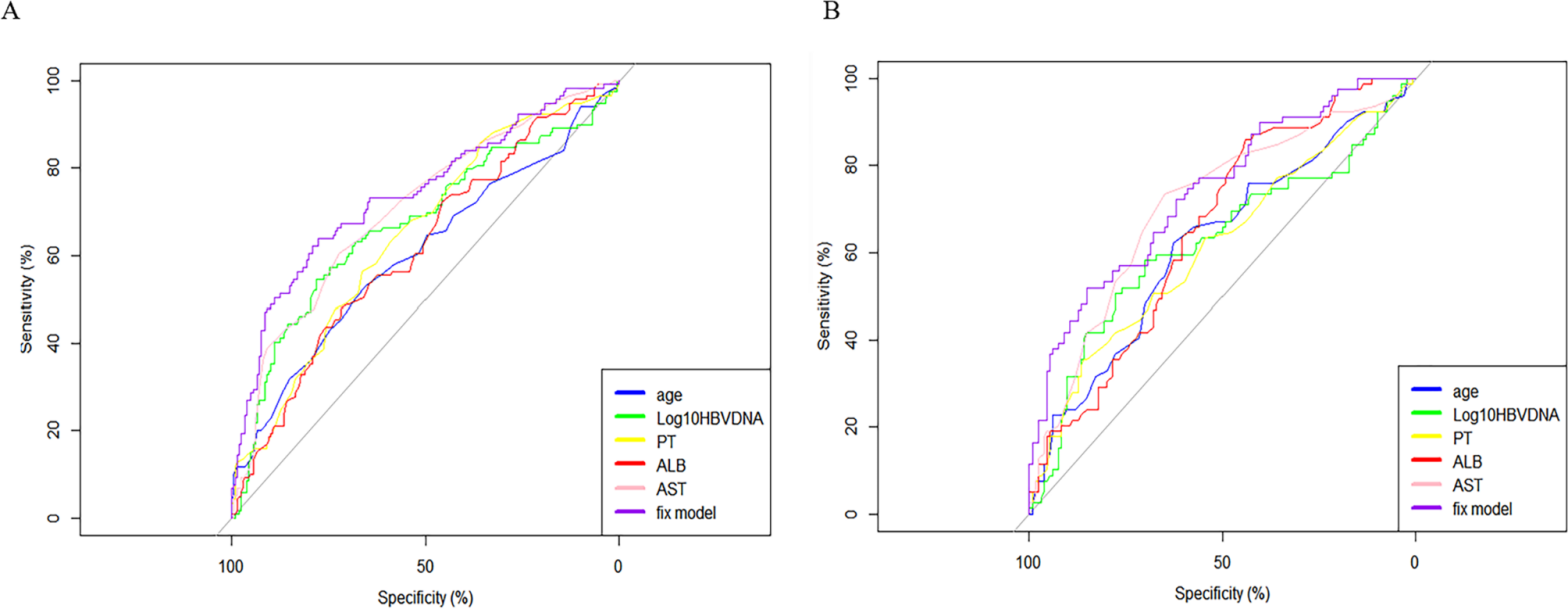
The ROC of invasive model for identifying significant liver inflammation in the training(A) and validation cohort(B) in the immune tolerate phase CHB patients. The predictive model for the training and validation cohort shown in Fig. 5A and B, the AUROC was 0.741 and 0.740, respectively.

We plotted the ROC curves to evaluate the performance of nomogram. Performance of the noninvasive model for predicting significant liver inflammation of IT-phase in the training and validation cohort are shown in Fig. 5. The ROC curve of the predictive model for the training and validation cohort shown in Fig. 5A and B, the AUROC was 0.741 and 0.740, respectively, higher than those for the independent factors.

## Discussion

Accurately assessing the severity of liver inflammation is crucial for the treatment decisions of CHB patients. The immune status of chronic hepatitis B is dynamic and is typically divided into four phases based on HBeAg, ALT, and HBV DNA levels. Due to the dynamic nature of the disease, monitoring the histological severity of the liver in patients during the IT phase is highly necessary. Therefore, we established and validated a non-invasive predictive nomogram model based on five serological markers (Age, AST, PT, ALB and HBV DNA) to facilitate the early identification of significant liver inflammation in the IT phase.

Despite numerous studies that have established non-invasive predictive models to assess the severity of liver inflammation^20,21^, including our previous study which identified AST, PT, GGT, and HBcAb as independent predictors of significant liver inflammation through multivariate logistic regression analysis^22^. Additionally, most research on IT-phase patients focuses on assessing the risk of liver fibrosis^23,24^. However, studies evaluating significant liver inflammation in IT-phase patients are relatively few, and they often lack model calibration and decision curve analysis.

In this study, we used LASSO regression analysis to select variables and reduce the risk of overfitting or underfitting due to confounding factors. Based on this analysis, we constructed a simple and intuitive nomogram. The accuracy of the nomogram was evaluated using DCA, calibration curves, and ROC curves. The model demonstrated good predictive performance for significant liver inflammation in the IT phase in both the training set and the validation set.

The IT phase is the earliest stage of chronic HBV infection, characterized by high serum HBV DNA levels, persistently normal alanine aminotransferase (ALT) levels, and little to no liver inflammation or fibrosis^11,25,26^. Currently, the treatment of CHB primarily focuses on patients with immune-active (IA) disease, cirrhosis, and decompensated cirrhosis. Generally, the IT phase is associated with a favorable prognosis, and antiviral therapy is not recommended in most guidelines because disease progression, including histological necrosis, inflammation, or fibrosis, is not active during this stage^4,6,11^. However, studies have reported that untreated IT-phase patients have a significantly higher risk of developing hepatocellular carcinoma (HCC), death, or requiring transplantation compared to treated immune-active patients^27^. Therefore, early identification and initiation of antiviral therapy are crucial for patients in the immune-tolerant phase.

Due to its invasive nature and associated complications, liver biopsy is generally not accepted for IT-phase patients. Serum ALT levels are one of the primary serological markers for evaluating liver inflammation and have long been considered the main indicator of liver inflammatory activity^28^. However, approximately 37% of CHB patients have normal or near-normal ALT levels but exhibit significant histological changes in the liver^29^. Thus, ALT levels do not fully reflect the severity of liver damage. Other studies have reported that AST levels have greater diagnostic value than ALT levels in assessing the severity of liver inflammation^30^. Therefore, there is an urgent need to develop a non-invasive model to predict significant liver inflammation in IT-phase CHB patients.

In this study, we identified Age, AST, PT, ALB and HBV DNA as independent predictors of significant liver inflammation in IT-phase patients. Our previous research found that AST levels significantly increased with the severity of liver damage^22^. AST is present in both the cytoplasm and mitochondria. Therefore, elevated AST levels indicate deeper liver damage and a higher likelihood of inflammatory infiltration and connective tissue formation, which may explain why AST is an independent predictor of significant liver inflammation^31^. Our study demonstrated that elevated serum HBV DNA levels are associated with significant liver inflammation in patients with CHB during the IT-phase, which is consistent with the study by Wu et al.^20^. Moreover, some studies have reported that elevated serum HBV DNA levels are closely related to the occurrence of HCC^27,32^. Therefore, antiviral therapy should be considered for patients with HBV infection exhibiting elevated HBV DNA levels and significant liver inflammation, regardless of ALT levels.

The PT was an independent predictor of liver significant inflammation. When liver function is impaired, inflammation and cell necrosis active the coagulation system, and consumption of coagulation-related substances in the liver triggers coagulation dysfunction. The PT reflects hepatocyte synthesis and is associated with a poor prognosis of significant liver inflammation^33^. A PT>5s that of the control value is prognostic of serious liver disease^34^. Additionally, age is an independent factor influencing significant liver inflammation. Following chronic HBV infection, liver inflammation tends to become more pronounced with increasing age. Moreover, age affects the natural course of CHB, with patients over 30–40 years old more likely to experience immune tolerance breakdown and liver inflammation. Studies have shown that individuals over 30 years have a significantly increased risk of developing HCC and HCC-related mortality^35^. Currently, many studies recommend considering antiviral therapy for CHB patients with a family history or cirrhosis or HCC after the age of 30–40 years^36–38^.

Our study also has some limitations. First, this is a retrospective study with a relatively small sample size, and prospective studies are needed for validation. Second, although this study is a multicenter study, due to the lack of available data, the genotypes of the patients were not assessed; most of the patients were of Asian ethnicity, so the model needs to be validated in other ethnic groups. Third, the immune status is dynamic, and the IT phase of patients might be temporary.

In conclusion, this study established a non-invasive model based on serological markers to predict significant liver inflammation in IT-phase CHB patients using a nomogram. This model helps reduce the need for clinical liver biopsies and facilitates the early identification and initiation of antiviral therapy in CHB patients with significant liver inflammation in the IT-phase.

## Electronic supplementary material

Below is the link to the electronic supplementary material.

Supplementary Material 1

## Data Availability

The datasets generated and analysed during the current study available from the corresponding author on reasonable request.
