# Supplementary material for: Noninvasive diagnosis model for predicting significant liver inflammation in patients with chronic hepatitis B in the immune-tolerant phase
Source: Sci Rep. 2025 Jan 24;15:3031. doi: 10.1038/s41598-025-87756-4 (PMC11760383; doi:10.1038/s41598-025-87756-4)
Supplement: Supplementary file 1 — Supplementary Material 1 [file 41598_2025_87756_MOESM1_ESM.docx]

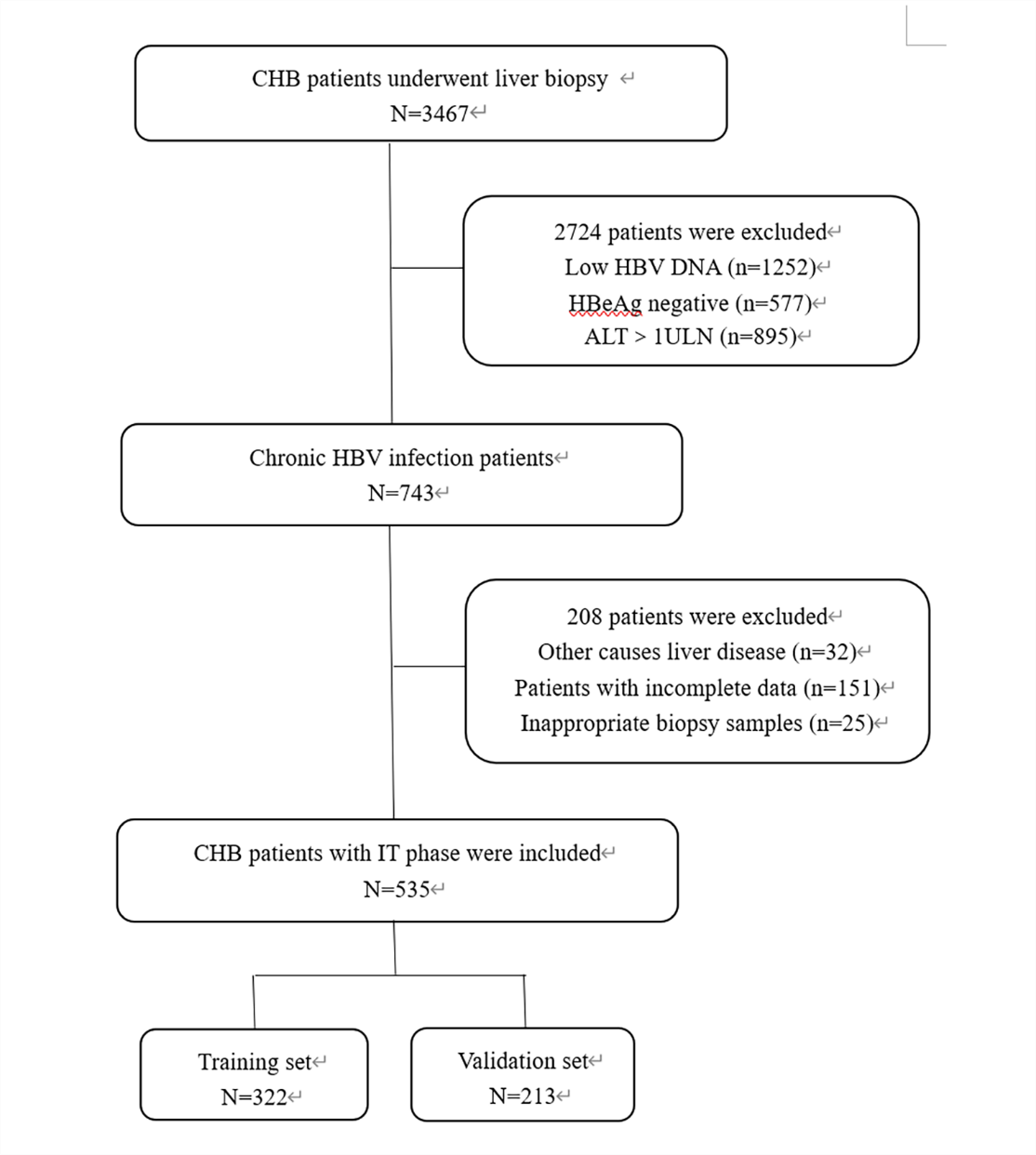


Figure supplemental material 1 Flowchart of the enrolment of patients with immune-tolerant phase. CHB, chronic hepatitis B; IT, immune tolerant; HBV, hepatitis B virus; ALT, Alanine aminotransferase;
